# The Protective Performance of Process Operators’ Protective Clothing and Exposure Limits under Low Thermal Radiation Conditions

**DOI:** 10.3390/biology11081222

**Published:** 2022-08-16

**Authors:** Ronald Heus, Boris R. M. Kingma, Birgit M. A. van Berlo, Douwe Mol, Hein A. M. Daanen, Kalev Kuklane

**Affiliations:** 1 Netherlands Academy of Crisis Management and Fire Service Science, Team Fire Service Science, Netherlands Institute for Public Safety, Zilverstraat 91, 2718 RP Zoetermeer, The Netherlands; 2 Human Performance Department, Unit Defence, Safety and Security, Netherlands Institute for Applied Scientific Research (TNO), Kampweg 5, 3769 DE Soesterberg, The Netherlands; 3 Department of Human Movement Sciences, Faculty of Behavioural and Movement Sciences, Vrije Universiteit Amsterdam, Van der Boechorststraat 9, 1081 BT Amsterdam, The Netherlands

**Keywords:** protective clothing, process operator, IR radiation, air gap, thermal protective performance, heat, skin temperature, exposure limit, petrochemical industry

## Abstract

**Simple Summary:**

Process operators have an important monitoring role in the petrochemical industry. In the event of process disruptions and incidents, a process operator is often the first responder and takes measures to diminish the effects of an incident. Thus, a process operator can be exposed to dangerous circumstances, and therefore personal protective equipment has to be worn. In case of fire, the operator may be exposed to high heat radiation levels. Previous studies have established maximum acceptable heat radiation levels for long- (>15 min) and short-term exposures (<5 min), these being 1.0 kW/m^2^ and 1.5 kW/m^2^, respectively. These limits were based on manikin measurements and physiological models. The validation of the protection of operators’ clothing in human trials is lacking. Therefore, twelve professional firefighters were exposed to three different heat radiation levels in process operators’ clothing. The experiments showed that the majority of the operators can be exposed for 5 min to 1.5 kW/m^2^, up to 3 min to 2.0 kW/m^2^, while exposure to 2.5 kW/m^2^ or above must be avoided. Due to a slower skin temperature rise, loose-fitting protective clothing was related to longer exposure times. We speculate that additional long-armed/legged (under)clothing may offer more protection and extend the exposure time to heat radiation.

**Abstract:**

During the early stage of a fire, a process operator often acts as the first responder and may be exposed to high heat radiation levels. The present limit values of long- (>15 min) and short-term exposure (<5 min), 1.0 and 1.5 kW/m^2^, respectively, have been set using physiological models and manikin measurements. Since human validation is essentially lacking, this study investigated whether operators’ protective clothing offers sufficient protection during a short-term deployment. Twelve professional firefighters were exposed to three radiation levels (1.5, 2.0, and 2.5 kW/m^2^) when wearing certified protective clothing in front of a heat radiation panel in a climatic chamber (20 °C; 50% RH). The participants wore only briefs (male) or panties and a bra (female) and a T-shirt under the operators’ clothing. Skin temperatures were continuously measured at the chest, belly, forearm, thigh, and knee. The test persons had to stop if any skin temperature reached 43 °C, at their own request, or when 5 min of exposure was reached. The experiments showed that people in operators’ clothing can be safely exposed for 5 min to 1.5 kW/m^2^, up to 3 min to 2.0 kW/m^2^, and exposure to 2.5 kW/m^2^ or above must be avoided unless the clothing can maintain an air gap.

## 1. Introduction

Process operators who are responsible for monitoring whether processes are successful can act as first responders in case of a starting fire incident and can, if necessary, take measures to prevent an incident from expanding. These are mainly found in occupational settings such as the petrochemical industry, the metal industry, industrial bakeries, firefighters, and military contexts. During a starting fire, closing a valve or placing an extinguishing monitor can be necessary activities, whereby an operator may be exposed to heat radiation. These first necessary actions can be carried out while waiting for the arrival of the firefighters who are better equipped to combat an incident. The fire service personnel have protective clothing [1] with a higher insulation value than process operators and also have independent respiratory protection.

This means that a process operator can be exposed to dangerous circumstances and that is only possible if the required personal protective equipment has been made available by the employer. Therefore, they need to be clothed in protective clothing against heat and flames, but with a limited level of protection compared to firefighters, because the process operators have to work full working days in that type of protective clothing, and adverse events involving flames and radiation heat happen much more seldomly.

The major aim of heat and flame protective clothing is to prevent skin burns from occurring. In the USA, the reported firefighter burn cases over 5 years were mostly related to areas outside of bunker gear (e.g., hands and head), interface areas (compatibility issues), or moisture, e.g., scalds [2]. An analysis of firefighter injuries for 2005–2007 by the US Fire Administration’s National Fire Incident Reporting System (NFIRS) showed that skin burns were mostly located around the head (39%), arm or hand (29%), neck or shoulder (18%), and leg or foot (9%) [3]. This was recently confirmed by Kim et al. [4], showing still most burn injuries are on the hands (36.9%), face and neck (33.9%), arms (10.4%), and upper body (7.5%). They also found that in most cases, the burn size was less than or equal to 1% of the total body surface area (TBSA).

Heat transfer to the human body through radiation in occupational settings commonly takes place through the clothing and the air gap [5]. The dry heat of the environment can be transferred to the body by convection, radiation, and conduction. Heat transfer also includes evaporation [6]. Compared to textile fibers, a still air layer has a lower thermal conductivity. When the air layer thickness exceeds 8–13 mm, dry heat and vapor exchange can take place due to natural convection within that air layer [7]. Moisture can laterally be distributed over the air gap area, which increases the evaporation of moisture and has a positive effect on heat loss, but a negative effect on the thermal protective performance of clothing [8,9]. Dry heat transfer through conduction can only take place when there is contact between the body and clothing, which mostly happens when the air gap is less than 7 mm [10,11].

While there is some evidence of the effect of air gap on skin temperature and burn injuries, most studies are done with a bench-scale testing apparatus [12,13], manikin tests [8,14], or modelling [15]. Those studies used these test methods to predict potential skin burns. At high radiation intensities (20–50 kW/m^2^), the time to a second-degree skin burn is within 5 s [16]. Due to the risks to the human body, human subject tests are never used at these high radiation intensities. Zhai and Li [16] concluded that these predictive methods are useful but can become invalid when adding protective fabrics between the heat source and skin. Using thermal manikins with mathematical models gives opportunities to overcome this, but also includes limitations. When using those test methods, the recommendation is to validate it with human testing [17]. Therefore, it is necessary to do human subjects’ tests at lower heat radiation levels.

In the nineties and 2000s, the Dutch applied research organization TNO conducted and published various studies into the ergonomics of firefighting clothing [18,19,20]. Test persons in different types of firefighting clothing were subjected to an ergonomic test battery, where exposure to heat radiation was one of the components. After analyzing these reports, it can be seen that new versions of firefighting clothing offered better protection against heat radiation than the earlier ones. Initially, the test persons did not tolerate prolonged exposure to the high heat radiation (tolerance time between 50 s and 2 min) due to pain sensation, but all firefighter suits in 2000 protected them so well that in almost all cases the test persons completed the planned exposure time of 2 min, with the measured skin temperatures varying from 34.9 °C to 37.8 °C. These values were well below the pain threshold of 43 °C.

In another study series [21], heat and moisture transfer under low- and high-frequency heat radiation through clothing materials with various moisture permeabilities was investigated with various technical means and on test persons. In related publications, the relationships between moisture transport, radiation levels, and clothing properties were clarified [22], and heat and mass transfer pathways were specified [23]. However, these studies did not focus on limit values under short-term extreme exposures, but on heat load and heat exchange under longer occupational exposures for avoiding or minimizing heat stress.

The other studies with test persons, e.g., by Heus and Kistemaker [18], established that firefighters can be exposed to a maximum of 7 kW/m^2^ for approximately 2 min, with the right protective equipment, without skin burns occurring. Due to the revision of the PGS 29 guideline [24], the current study aimed to determine experimentally whether the protective clothing of process operators in the petrochemical industry actually provides adequate protection at previously set limit values [25] and allows carrying out short-term actions of up to 5 min to prevent an incident from spreading rapidly without putting oneself at unnecessary risk while wearing less insulative protective clothing than what firefighters have. In addition to the safe values of 1.0 kW/m^2^ (>15 min) and 1.5 kW/m^2^ (<5 min) used to date, in this study, the test persons were exposed to 1.5, 2.0, and 2.5 kW/m^2^.

Besides measuring the maximum allowable exposure times to different radiation levels, it was essential to investigate the underlying factors that may cause limited exposure time. Next to the fabric properties, clothing fit is related to the thermal protective performance of clothing and the air gap between the garment and the skin [26], while layers of still air between clothing layers and skin contribute to thermal insulation [9]. Fit can also be different for different body parts, which indicates the importance of measuring the air gap at different locations of the body [27]. The influence of the air gap on protective performance has been studied extensively. With a novel method, 3D body scanning, the air gap in clothing can be measured up to 1 mm precisely [28]. An easier way to estimate the air gap, especially in field conditions, is by measuring the circumferences of the body and clothing, as done in the study of Chen et al. [8]. Therefore, besides the performance of the protective clothing itself, this study also investigated the effect of clothing fit on skin temperatures underneath the clothing at different body locations.

The main research question of this study was whether process operators can be exposed to higher radiation levels for a limited period of time (≤5 min) than the current set limit of 1.0 kW/m^2^ without exceeding the critical skin temperature of 43 °C. Simultaneously, the test persons were asked to rate their subjective responses at the different radiation levels.

Secondly, this study aimed to evaluate how the garment fit and how the air gap of operators’ clothing would affect the skin temperature change at radiation intensities in the range of 1.5–2.5 kW/m^2^. The hypothesis was that the thermal protective performance of operators’ clothing increases with air gap thickness between the body and clothing while moving.

As the process operators’ clothing came in two colors (dark blue and red), and discussions on color-related differences in protective performance are on the agenda, as a secondary outcome, the effect of color was also evaluated.

## 2. Materials and Methods

### 2.1. General

In this experimental validation study, test persons (TP) were exposed to different heat radiation intensities to determine, based on the rise in skin temperature, whether the existing safe radiation contours for process operators are adequate, too conservative, or too high.

### 2.2. Test Persons

The experiments were conducted with twelve (12) test persons, who were volunteers from the Gezamenlijke Brandweer (community fire services, GB). The test persons were all trained professional firefighters, as a requirement was that they must have experience with exposure to high temperatures. The characteristics of the test persons are listed in Table 1.

### 2.3. Ethical Considerations

Ethical approval was acquired from the internal ethics board at TNO (R2019-104). The test persons had been given information about the study earlier, and after filling in the health declaration, obtained approval from a medical doctor to participate in the study. They also signed the written consent to participate voluntarily in the study. The test persons arrived in the morning at the test location, were given a briefing on the procedures, and were shown the preparation room and test setup in the climatic chambers. They were also reminded about their right to quit the experiment whenever they felt the need for it.

### 2.4. Clothing

**Figure 1:**
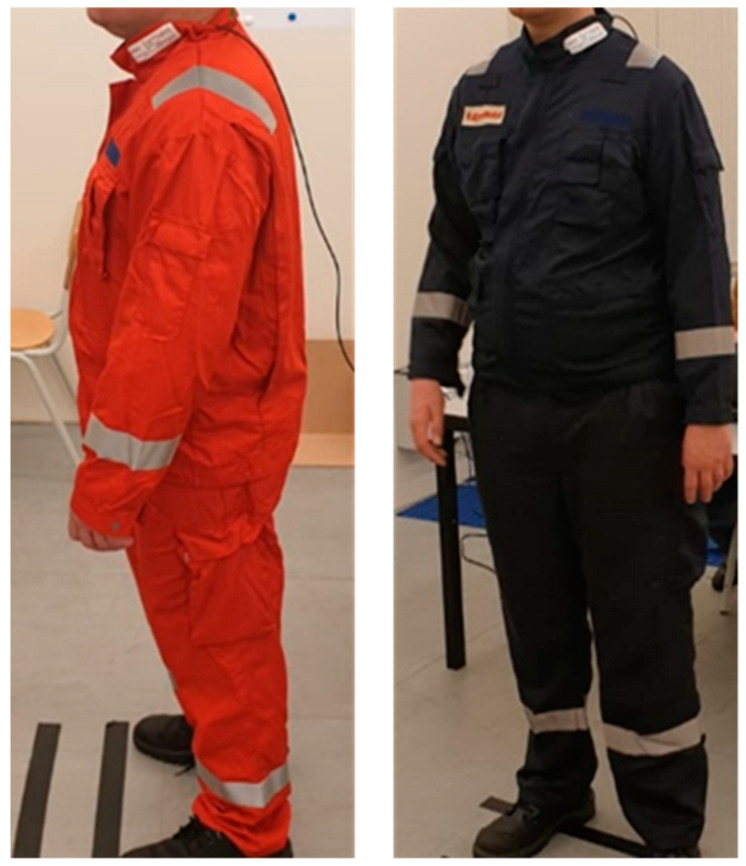
Protective clothing for process operators.

All experiments were carried out with used but clean clothing for process operators from ExxonMobil (Figure 1). The clothing material was Tecashield from TenCate, consisting of 93% Nomex^®^Comfort, 5% para-aramid, and 2% P140 (quality BV 185, weight 215 g/m^2^, construction 2/1 twill). The clothing, consisting of a jacket and trousers, was EN ISO 11612 certified [29]. All test persons wore the same clothing during all sessions. The clothing was supplied by ExxonMobil in two versions, namely dark blue and red (Figure 1). Under the operators’ clothing, all test persons only wore their own briefs/boxers (male) or panties and bra (female) and a T-shirt. The sizes of clothing had to be selected from the available ensemble sizes, thus the sizing was not standardized. The participants were provided with as close a proper size of clothing as possible. At the test site, they were provided a wide variety of clothing sizes that they could try on and select the ones that they considered most comfortable. The size of the trousers and jacket could be different and depended on the best fit for the test persons.

### 2.5. Study Design

All experiments were performed in one day in the TNO climate chambers in Soesterberg. The test persons were exposed to heat radiation from an infrared radiation panel three times for a maximum time of 5 min using a Latin Square design. Since sweating is not yet initiated within 5 min exposure [30], it was neglected in this study. The time between two sessions for one test person was approximately 2 h. Between the tests, the test persons sat quietly in a waiting room at a constant room temperature of about 20 °C.

#### 2.5.1. Test Setup

**Figure 2:**
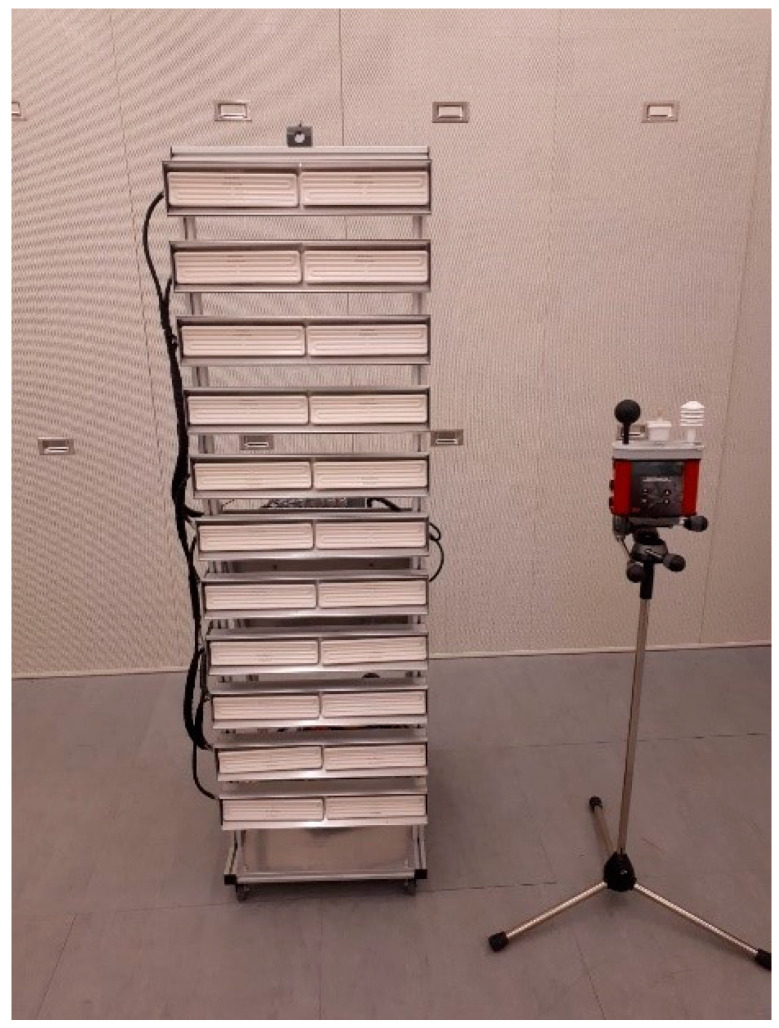
Radiation panel.

The test persons were exposed to heat radiation generated by a system with 11 × 2 infrared (IR) panels (Elstein FSR 400, à 6.0 × 24.5 cm, Figure 2). The dimensions of the whole panel system were 150 × 50 cm, and the bottom edge of the lowest panel was located 21 cm above the floor. Each panel emits a maximum of 25.6 kW/m^2^ (500 °C at 400 W) at a beam angle of 90° (ceramic infrared panel radiators).

By varying the distance of the test persons from the panel system, different radiation intensities could be achieved. The correct distances were determined in earlier tests with a WBGT measuring instrument (Figure 2; WBGT is a weighted average of the globe temperature, the air temperature, and the natural wet bulb temperature, where the black globe temperature reflects the temperature experienced by people as a result of heat radiation—see ISO 7243 [31]—because the (black) bulb temperature is related to the radiation intensity). Thermal radiation levels were estimated by iterating heat radiation for calculating the black bulb temperature according to Liljegren et al. [32] under the assumption that the black bulb is radiated directly by the panel (cosine of zenith angle = 1 and fraction of direct radiation = 1).

**Figure 3:**
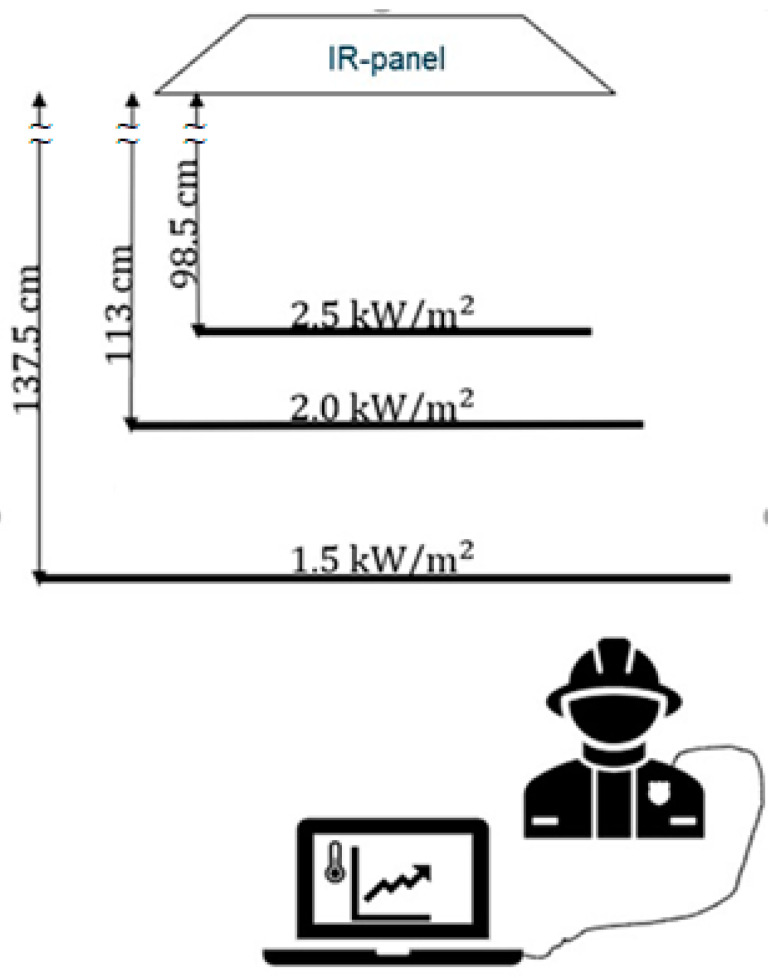
Measurement setup.

The actual distances can be found in the schematic overview of the measurement setup in Figure 3. To prevent climate differences between conditions other than the imposed intensity of infrared radiation, the experiment took place in a climate chamber set at an ambient temperature of 20 °C and 50% relative humidity. The test persons were equipped with skin temperature sensors (thermistors) connected to an MSR145W logger (range −55 to +125 °C, accuracy within +5 to +45 °C ± 0.1 °C, MSR Electronics, Switzerland) for the continuous registration of skin temperatures at the chest, abdomen, upper arm, thigh, and knee, and wore the process operators’ normal protective clothing including a helmet and sunglasses. Although in practice, radiation can come from all around, for the purpose of this safety study, the choice was made for radiation from the front only, which can be also most realistic during an incident when trying to put out a fire. Therefore, all skin temperature sensors were placed facing the front.

#### 2.5.2. Test Procedure

The first session started with selecting the clothing set and measuring the height and weight of the test persons. Prior to each session, the temperature sensors were attached to the skin using air and water vapor permeable tape (Fixomull Stretch). Subsequently, prior to exposure, the subjective experience of temperature, comfort, pain, and perceived exertion [33,34] were determined (Table 2).

**Figure 4:**
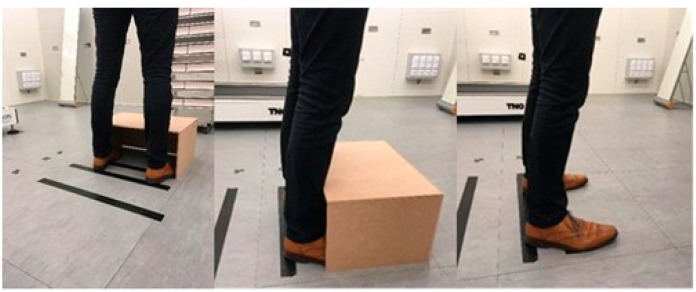
Instructional pictures on methodology for consistent placement of the test person relative to the radiation panel.

Subsequently, after entering the climate chamber, the MSR data logger was connected to a laptop with a cable allowing the real-time monitoring of the skin temperature. The test person was then instructed to make shin contact (Figure 4) with an inverted U-shaped wooden box that was precisely aligned at a specified distance depending on the exposure condition, while the test person stood upright in front of the radiation panel. Subsequently, the box was removed, and the test persons had to make stepping movements while the toes were not allowed to leave the ground to fix the test persons in the right spot. Then, the reflective screen between the test person and the working radiation panel was removed so that the specified heat was instantly applied to the test person. The exposure time was set to a maximum of five minutes. The criteria for stopping earlier were: a skin temperature at one of the measured locations above 43 °C or a request from the test person that it is no longer acceptable to stay in front of the radiation panel, for example, due to pain. During the last 30 s of a session, or immediately after the termination of the test (if skin temperature at any measured spot reached 43 °C or the test person requested to stop) the participants were asked again for their thermal, comfort, and pain sensations, and perceived exertion.

### 2.6. Air Gap Estimation

The air gap between body and clothing was measured after exposure to the radiation panels. This was realized by measuring the circumferences of the body and clothing. While the participants were wearing the protective clothing, marks were made on the clothes at the skin temperature sensor locations. Then, the clothes were taken off and put on the floor. At the level of the marked locations on the clothing and the location of the temperature sensors on the skin, the width of the clothing or circumference of the body part was measured, respectively. The chest sensor often happened to be located underneath the edge of the pocket, outside, or underneath the pocket depending on the test person and clothing size. Those different layers of clothing above these sensors could influence the skin temperature measured at the chest. The pockets located at the chest can be seen in Figure 1.

For the chest-level jacket circumference, a different procedure was followed. The height of the mark was at the height of the axilla. At that point, the arms’ seams were connected to the core of the clothing. The width of the clothing was measured up to the arms’ seams. The measurer stated that on both sides, roughly 4 to 7 cm were missing due to this procedure at chest level. Therefore, the values of the circumferences of the clothing at chest level were corrected. On both sides, 4 to 7 cm were missing while measuring the width of the clothing. This means 16 to 28 cm was missing for the circumference of the chest depending on the clothing size. An interpolation was made between the largest and smallest size and a value between 16 and 28 cm was added to the jacket’s chest circumference. The corrected values for the circumferences of the body and clothing were used to make an estimation of the air gap between body and clothing.

For 11 participants, the circumferences of the clothing were larger compared to the circumferences of the body, except for participant 5 at the level of the abdomen. This could be explained by the stretching of the clothing or compression of the tissues. For this case, the assumption of no air gap at all was made and the air gap was set to zero. For all other measurements, the mean air gap between body and clothing was calculated.

Assuming a circular cross-sectional area of the body and surrounding garments, the mean air gap thickness between body and clothing was calculated with the following equation:

(1) $$Air\;gap\;thickness=\frac{Cc-Cb}{2\pi },$$

where *air gap thickness* is the mean distance between body and clothing in mm, *Cc* is the circumference of the clothing section in mm, and *Cb* is the circumference of the body part in mm. The air gap was calculated separately for the four different body locations (chest, upper arm, abdomen, and thigh). The mean air gap of all body locations was calculated to estimate the overall air gap of the whole suit. The mean air gap of the chest, upper arm, and abdomen was calculated to give an estimation of the air gap of the jacket. The circumferences of body and clothing were not measured at the knee. The mean air gap of the thigh represented an estimation of the minimal air gap of the trousers.

### 2.7. Data Analysis

#### 2.7.1. Data Analysis for Exposure Limits

Basic statistics were performed with Excel. For each radiation intensity data range (minimum, maximum values), mean, median (middle observation), and standard deviations of the exposure duration, skin temperatures, and subjective responses were determined. Differences in maximum exposure time and the subjective measurements between the radiation intensities were tested with repeated *t*-tests and the results were considered significant at *p* ≤ 0.05.

#### 2.7.2. Data Analysis and Statistics for Evaluation of the Air Gap

The skin temperature was measured every second, after which the temperature change over the exposure time (∆Tskin/t in °C/s) was calculated. This provided an estimation of the rate of skin temperature development; the change in skin temperature over time. The test persons moved away from the radiation source after five minutes of exposure or after the skin temperature had reached 43 °C. The measurement of the skin temperature continued after removing the radiation source. This continuation of measurement was not considered with the calculation of the ∆Tskin/t. For every radiation level, body location, and participant, a linear regression line was made between the skin temperature and time in SPSS. The slopes of the regression lines represent the ∆Tskin/t. The goodness of fit of the regression line was estimated to test whether the ∆Tskin/t can be used as a valid variable. The R^2^ gives an indication of the percentage of the variance the regression line explains. An R^2^ of 0.90 means that 90% of the variance is explained with the regression line.

For the first part of the statical analysis, the participants were divided into three groups with 4 persons in each: relative tight fit, relative regular fit, and relative loose fit. Firstly, groups were made for all local air gaps separately. For example, the participants were equally divided into three groups: relative tight fit at the chest, relative regular fit at the chest, and relative loose fit at the chest. The same procedure was used for the measurements at the abdomen, upper arm, and thigh. Secondly, groups were made regarding the fit of the jacket. The averages of the air gap of the chest, abdomen, and upper arm were used to divide the participants into the three groups (relative tight, relative regular, and relative loose fit of the jacket). Thirdly, groups were formed for the entire garment (jacket and trousers). The averages of all measured air gaps (chest, upper arm, abdomen, and thigh) were used to estimate the air gap of the entire garment. The analysis of variance (ANOVA) test for the comparisons between fit groups was carried out with SPSS to analyze the difference in group means of exposure time and ∆Tskin/t. To test whether the air gap had an effect on the subjective measurements (RPE, comfort sensation, temperature sensation, and pain sensation), ANOVA tests were conducted. These tests were conducted for each subjective measurement separately because the responses are interrelated. For example, when thermal sensation reaches the highest values, then this goes over to a pain sensation, and both could affect the comfort sensation.

A disadvantage of using ANOVA was the small group of participants in this study. Therefore, the second part of the statistical analysis consisted of regression analysis. Before running the regression analysis, correlations between all measured variables were tested. The regression analysis was performed to obtain simple predictive models between air gap thickness and skin temperature development or exposure time. Simple regressions were performed multiple times with all different air gaps as independent variables and exposure time or ∆Tskin/t as dependent variables. To test whether there were other contributing secondary factors, a stepwise multipredictor linear regression analysis was conducted with the significant predictors obtained with the simple linear regression analyses. The variables with significant correlations with exposure time or ∆Tskin/t were inserted in the multipredictor linear regression analysis.

## 3. Results

### 3.1. Exposure Time

The percentiles of the tested population whose skin temperature stayed under 43 °C for 1, 3, and 5 min under the tested radiation intensities are shown in Table 3. It can be seen that even under the lowest tested radiation, not all could continue the exposure for the full 5 min before either their skin temperature at the measuring point reached 43 °C or they themselves decided to stop (it was assumed that the pain threshold was 43 °C, too).

**Figure 5:**
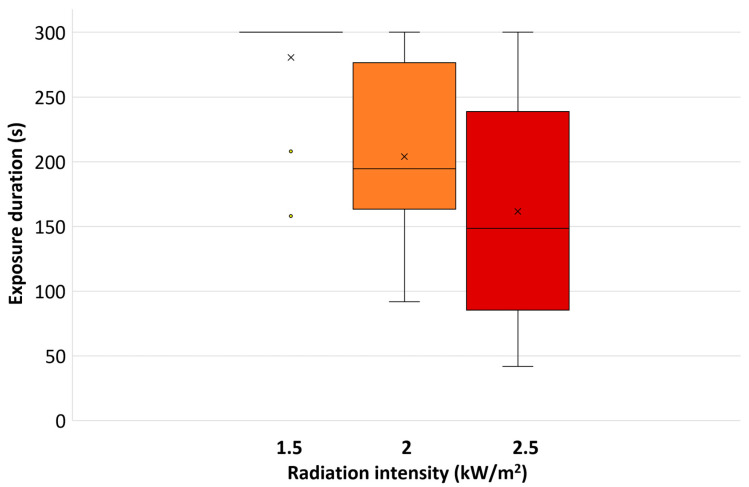
The median (horizontal line) and mean (×) exposure times with the standard deviation and the outliers (◦) at the different radiation intensities.

The median and mean exposure times with the standard deviation and the outliers at the different radiation intensities are shown in Figure 5. The median values for the exposure time for the radiation intensities of 1.5, 2.0, and 2.5 kW/m^2^ were 300, 195, and 149 s, respectively. Due to two test persons who did not reach the maximum exposure time at 1.5 kW/m^2^, the average exposure time was slightly lower than 5 min, while the median for this radiation intensity was 5 min. The exposure time at 1.5 kW/m^2^ was significantly higher than that for 2.0 and 2.5 kW/m^2^ (*p* < 0.005 and *p* < 0.0005, respectively), while the latter two did not differ significantly from each other (*p* > 0.2).

### 3.2. Skin Temperature

**Figure 6:**
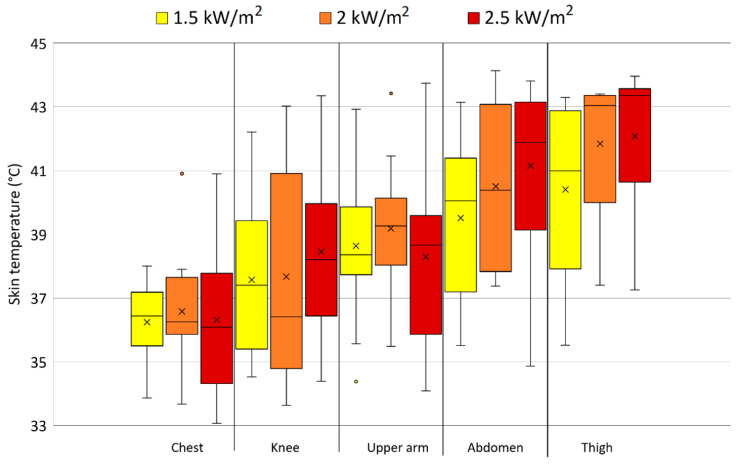
The median (horizontal line) and mean (×) highest measured skin temperatures with the standard deviations and the outliers (◦) at the various body locations across the test persons at the different radiation intensities.

The median and mean highest achieved skin temperatures with the standard deviation and the outliers at the various skin locations across the test persons are shown for each radiation intensity in Figure 6. On average, the skin temperatures for all exposure levels remained below the critical value of 43 °C, but individually, with the exception of chest temperature, temperatures of 43 °C or higher were measured. It has to be considered that the experiments were stopped when the temperatures reached 43 °C, and much higher values were not possible, except for some after-rise related to the thermal inertia of the clothing system [35]. The highest skin temperature measured in this study was 44.1 °C.

**Figure 7:**
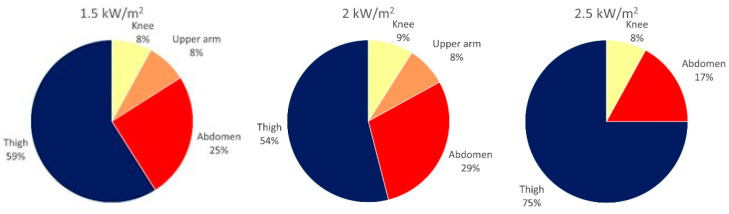
The ratio of skin locations with maximum skin temperatures.

Figure 7 shows the ratio of skin locations with maximum skin temperatures. As can be seen, the most affected areas under all radiation intensities were the thighs and abdomen, and this can be directly related to the minimal air gap—or reduced air gap due to the stepping motions—between the textile and the skin. Although the test persons were set in a fixed position, the most protruding body parts with minimal ventilation and air gap were the abdomen and thighs. This could be another reason for the highest temperatures at these locations. At the chest, the highest temperature was never measured. This could be explained by the fact that the chest sensor might have been covered by multiple textile layers of the chest pockets.

**Figure 8:**
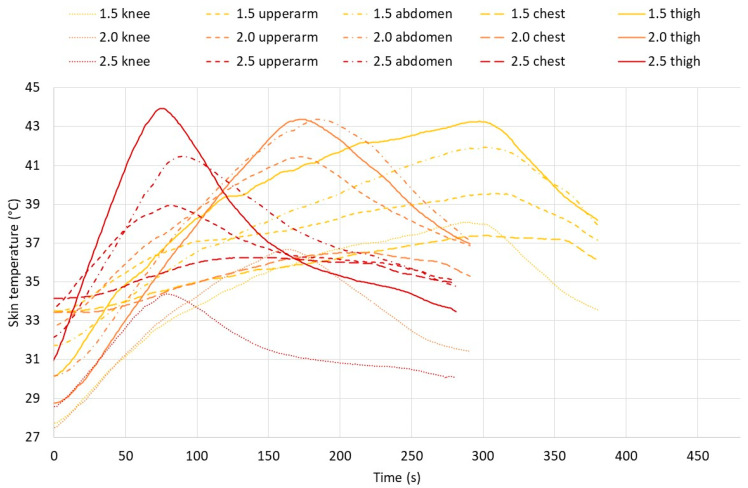
Skin temperatures (knee, upper arm, abdomen, chest, and thigh) of TP6 during exposure to the different radiation intensities. TP6’s exposures were terminated at 300, 166, and 76 s at 1.5, 2.0, and 2.5 kW/m^2^, respectively.

Figure 8 shows the typical temperature trends at the different skin locations over time of a test person (TP6). The graph shows that this test person was exposed for the full 5 min at 1.5 kW/m^2^. This test person had to stop earlier at 2 and 2.5 kW/m^2^ due to reaching a skin temperature of 43 °C (thigh). The individual curves for different test persons could differ, while the general trend followed the temperature change pattern seen in this participant.

#### Skin Temperature Change

With the skin temperatures from t = 0 till the end of the heat exposure, the ∆Tskin/t was calculated with the use of a regression line between skin temperature and time in SPSS. The results are shown in Table 4.

The correlation was used to check ∆Tskin/t results (R^2^). A higher radiation intensity was related to an increase in skin temperature development. The regression line of TP5 at a radiation level of 2.5 kW/m^2^ at the chest showed an R^2^ of 0.019. TP5 quit after 42 s and had the lowest tolerance times of all participants. All other regression lines of all participants showed an R^2^ between 0.754 and 0.998.

The ∆Tskin/t significantly increased together with the increase in radiation intensity at the abdomen (*p* = 0.009), thigh (*p* = 0.007), and knee (*p* = 0.007). Posthoc tests showed that the difference in ∆Tskin/t was significant between 1.5 kW/m^2^ and 2.5 kW/m^2^ for all three skin locations. There was no significant increase at the chest (*p* = 0.096) and upper arm (*p* = 0.115).

### 3.3. Subjective Responses

The thermal, comfort and pain sensations, and perceived exertion (RPE) are shown in Table 5.

The initial values of the thermal sensations were neutral and the same in all tests. The thermal sensation at the end of the exposure at 1.5 kW/m^2^ differed significantly from the thermal sensation at 2.5 kW/m^2^. The response between other intensity levels did not differ significantly.

All test persons felt comfortable prior to exposure. The discomfort at the end of the exposure period ran from “(slightly) uncomfortable” to “very uncomfortable”. The discomfort increased as the radiation intensity was higher, but the differences were not significant.

There was no pain present prior to exposure. The average pain perception after the exposure varied from “no pain” to “somewhat painful”. Although a slightly increasing trend can be seen as the radiation intensity increased, there were no significant differences between the different radiation intensities.

The test persons were at rest beforehand and experienced no effort. The effort afterward was experienced as “very light” to “light”. There were no significant differences in perceived exertion between the different exposure levels.

### 3.4. Air Gap

The results of the calculated air gaps between body and clothing are shown in Table 6. The means of the air gaps at the chest, upper arm, abdomen, and thigh give an estimation of the overall fit of the whole suit. The means of the air gaps at the chest, upper arm, and abdomen give an estimation of the fit of the jacket.

#### 3.4.1. Comparisons between Groups: Tight, Regular, and Loose Fit

A series of one-way ANOVAs were conducted with exposure time or ∆Tskin/t as the dependent variable, and the (relative) clothing fit groups as the independent variable. The ANOVAs were conducted separately for all three radiation intensities. To test whether this effect is specifically related to the different body parts, the groups were made several times and covered each skin temperature location, jacket, and whole set (see Table 7). The thigh area was used to represent trousers. To test the differences in group means for exposure time, a one-way ANOVA test was conducted. The outcomes are shown in Table 7. The outcomes of these tests give an indication of the differences between group means in exposure time.

**Figure 9:**
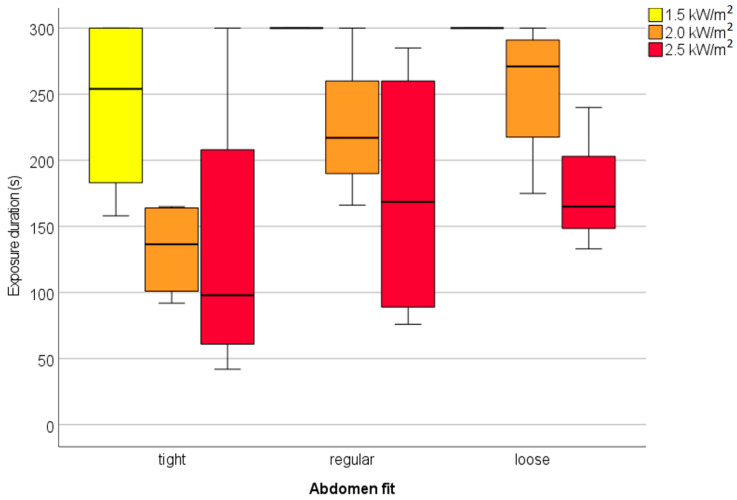
Differences between tight, regular, and loose clothing fit groups in exposure time, measured at three different radiation levels. In this figure, the tight fit group consists of 33.3% of the participants with the smallest air gap at the abdomen, the next 33.3% in the regular fit group, and 33.3% of the participants with the largest air gap at the abdomen in the loose fit group. The upper and lower ends of the boxes represent the 25th and 75th percentiles. The line inside the box marks the median. The bars represent the values within 1.5 times the interquartile range of the box.

The results in Table 7 show a significant difference in group means based on the clothing fit around the abdomen at 2.0 kW/m^2^. The ANOVA with a posthoc Tukey HSD shows a significantly lower exposure time for the group with relatively tight-fitted clothing around the abdomen, compared to relatively loose-fitted clothing around the abdomen. In Figure 9, the significant difference at the radiation level of 2.0 kW/m^2^ is graphically illustrated. No other significant differences in group means for exposure time were found with the one-way ANOVA tests. Figure 9 also shows the clipping effect at the radiation intensity of 1.5 kW/m^2^. In the regular and loose fit group, all participants reached the maximum exposure time of 300 s.

The same one-way ANOVAs were conducted with the ∆Tskin/t as the dependent variable. The outcome gave an indication of the differences between group means in the ∆Tskin/t. Groups were made several times and compared between the specific ∆Tskin/t corresponding with the body location-specific groups. The results of the one-way ANOVAs showed no significant differences between group means in ∆Tskin/t.

#### 3.4.2. Effect of Clothing Fit on Change in Subjective Responses

The difference in scores on the subjective variables between the start and the end of the measurement was inserted in the one-way ANOVA. No significant differences were found between groups in ∆RPE. At the radiation intensity of 2.5 kW/m^2^, significant differences were found between group means in comfort sensation with groups based on the clothing fit at the upper arm (F(2,9) = 5.955, *p* = 0.023) and at the thigh (F(2,9) = 5.955, *p* = 0.023). Significant differences in temperature sensation with groups based on the clothing fit at chest (F(2,9) = 5.740, *p* = 0.025), jacket (F(2,9) = 6.248, *p* = 0.020), and whole suit (F(2,9) = 4.993, *p* = 0.035) were found at the radiation intensity of 2.5 kW/m^2^. At the radiation intensity of 2.0 kW/m^2^, a significant difference between group means in temperature sensation was found with groups based on the fit of the jacket (F(2,9) = 7.620, *p* = 0.012). A higher temperature sensation change was found for all three clothing fit groups at 2.5 or 2.0 kW/m^2^. For groups based on the fit at the chest, a significant difference was found in pain sensation at 1.5 kW/m^2^ (F(2,9) = 0.978, *p* = 0.011). The homogeneity of variances was violated because all participants in the relatively loose-fit group did not report any sensation of pain.

#### 3.4.3. Regression Analysis of Clothing Fit on Skin Temperature Change

Regression analysis was performed because of the small groups and the clipping effect in the first part of the statistical analysis, the ANOVAs. Before running the regressions in SPSS, the assumptions of the regression analysis were checked. The normality of all variables was checked. Some data were not normally distributed, therefore a robust method was used for the regression analysis, with the use of a bootstrapping function before running the regression analysis to avoid bias. The outcome variable exposure time at 1.5 kW/m^2^ did not meet the assumption for normality as well as linearity with other variables. This could be explained by the lack of variance in this variable, which caused a clipping effect in the data. Ten of twelve participants reached the maximum exposure time, 300 s. Correlations between the measured variables were checked.

Multiple simple linear regression analyses were conducted with exposure duration or ∆Tskin/t as the dependent variables. All different air gaps were inserted one after another as independent variables. The R^2^ shows the proportion of variance explained by the model. The b-value shows the gradient of the regression line. These results could be interpreted in the following way. For example, with the exposure time as the dependent variable and the air gap at the thigh as the independent variable, a significant regression equation was found (F(1,10) = 6.032, *p* = 0.05), with an R^2^ of 0.379. Participants’ exposure time at a radiation level of 1.5 kW/m^2^ was equal to 203.758 + 3.020 (air gap at the thigh) seconds when the air gap was measured in mm. Participants’ average exposure time increased by 3.02 s for each mm increase in the air gap.

The results, the *p*-values of the regression analyses, are summarized in Table 8. The significant outcomes of the simple regression analyses indicated that the air gaps explain a part of the variance in the dependent variable (∆Tskin/t and exposure time). At the radiation intensity of 1.5 kW/m^2^, 16 significant regression analyses were found. At the radiation intensity of 2.0 kW/m^2^, 13 significant regression analyses were found. At the radiation intensity of 2.5 kW/m^2^, only one significant regression analysis was found with the ∆Tskin/t of the whole suit as the dependent variable and the air gap at the upper arm as the independent variable. No significant regression outcomes were found with the air gap at the chest as the independent variable. A larger air gap commonly showed a positive relation with exposure time and a negative relation with skin temperature change over time.

#### 3.4.4. Regression between Skin Temperature and Anthropometric Parameters

Besides the air gaps and Tskin at t = 0, body length, body weight, and BMI showed significant correlations with exposure duration and ∆Tskin/t. Body weight showed the most and the highest correlations with exposure duration and ∆Tskin/t. Because of the intercorrelation between the three variables, only body weight was inserted in the multiple predictor regression analysis. Simple linear regression analyses with exposure time or ∆Tskin/t as outcome variables and body weight as predictor variables showed significant outcomes. However, including body weight in a stepwise multiple predictor regression analysis together with the air gap did not result in significant prediction models. Adding body weight to the regression model did not result in a stronger model.

### 3.5. Effect of Color

**Figure 10:**
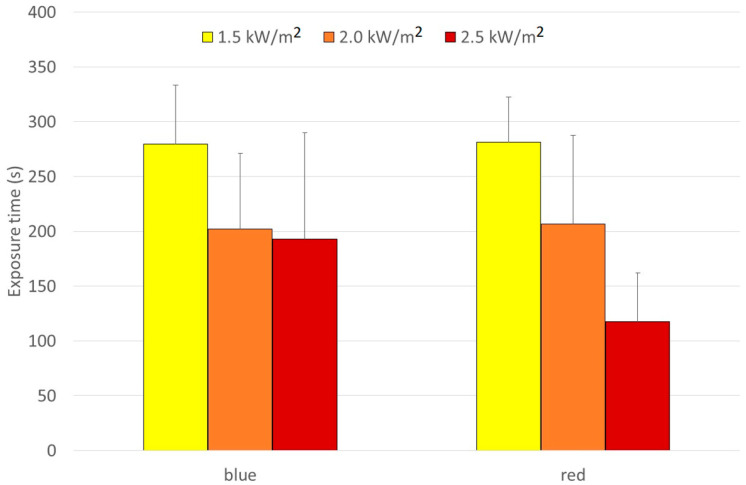
Mean exposure time in blue and red clothing at different radiation levels.

There were no observations of any significant differences caused by color at 1.5 and 2.0 kW/m^2^, while at 2.5 kW/m^2^, the exposure time in blue protective gear was significantly longer than in the red one (Figure 10). The ∆Tskin/t pattern confirmed this result.

## 4. Discussion

### 4.1. Exposure Limits

The validation study on exposure to heat radiation conducted with test persons showed that the previously set limit value of a maximum of 1 kW/m^2^ [25] is a conservative estimate for operators. This estimate was made on the basis of previous studies with thermo-physiological computer models [36] and a thermal manikin [37]. In the current study, it appeared that the majority of test persons, with two exceptions (both in the tight-fitting group and one of them wearing the most tight-fitting clothing set, see Figure 9), had no problem whatsoever with an exposure of 5 min to 1.5 kW/m^2^. This is also in line with the safe heat radiation contour of 1.5 kW/m^2^ [38].

In this experiment, the exposure was stopped at the pain threshold (Tskin = 43 °C or actual pain occurring = subjects stopped themselves). Considering physical danger-related stop criteria, i.e., the risk of a first-degree burn injury, the skin temperature could be higher or exposure longer [39], as the rate of skin temperature increase at the radiation intensity of 1.5 kW/m^2^ was relatively slow (Figure 8 and Table 4) unless tight-fitting clothes were worn (Figure 9). Exposure to 2.5 kW/m^2^, however, contributes to a much quicker skin temperature change (Figure 8 and Table 4) that may within less than half a minute increase the skin temperature to the level where first- or even second-degree burn injuries can occur.

The test persons in this study wore only briefs/boxers and a T-shirt under their operators’ clothing. This allowed direct contact with the protective clothing on parts of the bare skin. The temperature sensors on the chest and abdomen were covered with undergarments. Despite this extra protection, in some cases, temperatures above 43 °C were measured on the abdomen. A possible cause of this rise in skin temperature is that the clothing was relatively tight at the level of the abdomen and the (insulating) air gap was, therefore, less than at the level of the chest, or protruded closer to the radiation panel than the chest, where no critical skin temperatures were measured. The chest (sensor) was covered by the pockets as additional layers. Sufficiently loose clothing and possibly a long underwear layer can contribute to operators being (even) better protected against high heat radiation levels. Moreover, textile or clothing constructional elements that keep spacing between the protective layer and the skin would give an increased protective effect.

Extra insulating layers can enable operators to perform work even at 2.0 kW/m^2^. On the basis of the current experiments, it was found that without additional undergarments, people could be exposed to this heat radiation for at least three minutes. At 2.5 kW/m^2^, the acceptable exposure time is too short to be able to quickly take action in the event of a fire incident.

### 4.2. Clothing Fit and Air Gap Effects

The participants were divided into three groups with relatively tight, regular, and loose-fitted clothing. Except for the chest (effect of a pocket over the temperature sensor) and somewhat less for the upper arm, ∆Tskin/t vs. clothing fit showed higher variation and larger temperature change for a tight fit, while regular- and loose-fit groups had approximately similar variation and an only slightly lower temperature change for a loose fit. Due to the small number of test persons in each group, the results are not conclusive. However, this result complies well with reported technical measurements on the sizing of cold protective clothing [40] and footwear [41] insulation, where a larger size did improve the insulation up to about 4% compared to the correct size, while a size or two smaller provided much lower insulation due to less air gap and/or the compression of the insulation layers. However, too-loose clothing will not increase insulation much further, but can instead introduce other risks, such as, for example, hindrance from the oversized clothing or the risk of getting caught in machinery.

The air gap at the abdomen showed a significant effect on the exposure duration at 2.0 kW/m^2^. The variable *air gap at the abdomen* showed the largest variance compared to the other measured air gap locations within the clothing fit groups. The participants were asked to make stepping movements during exposure that could cause a pumping effect of the air gap at the thigh. The arms were moving slightly, due to the stepping movement. Therefore, the pumping effect could also be slightly present at the upper arm and even in the upper body. The air gap at the abdomen seemed to be the most stable air gap, with enough variance in air gap over all participants, which could explain the significant findings for the effect of air gap on exposure duration at the radiation intensity of 2.0 kW/m^2^. The lower (1.5 kW/m^2^) and higher (2.5 kW/m^2^) radiation intensities did not show air gap effects on exposure duration. At the lowest radiation intensity this could be explained by the clipping effect (exposure length was maximally 5 min). At the highest radiation intensity, the exposure times were relatively short. At this radiation intensity, the process operators’ clothing did not seem very suitable. This could be related to the air gap thickness, but also the fabric thickness or the backside emissivity that can have a large influence on the thermal protective clothing, which could be too low to protect against this level of the highest used radiation intensity [15].

Chen et al. [8] studied various fabrics and found that the thermal insulation increases with the thickness of the air gap with all fabrics. Heat transfer through conduction is not possible when there is an air gap present. As the air gap increases, the heat transfer to the skin decreases. A critical point was observed above, which is that natural convection is not sufficient for thermal insulation. This indicates the existence of an optimum air gap thickness. The critical point was found at 10 mm. The rate of increase in thermal insulation gradually decreases as the air gap becomes thicker before reaching the optimum air gap. This was also well demonstrated by Psikuta et al. [15]. When the air gap exceeded the optimum thickness, the rate of thermal insulation increase showed a smaller decrease. Song [14] tested different sizes of single-layer garments with a manikin at high radiation levels (83 kW/m^2^) and used 3D body scanning to measure the air gap thickness. The results of the manikin tests showed a higher risk for burn injuries with smaller air gap thickness. The model predicted an optimum air gap of around 7–8 mm. After reaching the optimum air gap, the thermal protective performance of the single-layer clothing seemed to decrease. The results of the present study did not indicate optimum air gap size.

The findings of this study on the effect of the air gap at the abdomen were confirmed with the regression analyses. With the regression analyses, more air gap locations were found as significant predictors of the exposure time and skin temperature change over time compared to the statistical analysis between groups. This difference in significant outcomes between the two kinds of statistical analyses can be explained by the small number of participants and the ratio scale, which is not considered in the comparisons between groups. The results of the regression analyses showed that a larger air gap, especially at the level of the abdomen and upper arm, is related to an increase in exposure duration and a decrease in skin temperature change over time at a radiation intensity of 1.5 and 2.0 kW/m^2^. This is in line with the hypothesis of this study, that an increase in air gap thickness is related to an increase in the thermal protective performance of process operators’ clothing and confirms multiple other studies related to the air gap and clothing fit [8,12,14,42,43]. At the radiation intensity of 2.5 kW/m^2^, only the air gap at the upper arm seems to be an indicator of the mean skin temperature change over time of the whole body.

A limitation of the procedure for estimating the air gap between the body and clothing in this study was that it assumed that the air gap was a stable air gap and equally divided around the body. This assumption can contain mistakes because folds and contact areas are not considered [7]. The posture of the body and body movements could also affect the size of the air gap [5]. Three-dimensional body scanning can give a more accurate determination of the air gap thickness and the contact areas between body and clothing, which is recommended for use in further research [28]. Simultaneously, measuring circumferences and calculating the average air gap is a useful method, especially for quick evaluations of protective clothing, and is also suitable in field trials. Another limitation of this part of the study was the relatively small number of participants (12) for the statistical tests with three clothing fit groups of four participants each. The ratio scale was not considered for the statistical test with three groups (relatively tight, regular, and loose fit). For an optimal study, it would be better to compare the same participants, under the same circumstances with tight, regular, and loose-fitted clothing, as was carried out in a manikin study by Veselá et al. [43]. The same limitations can also be brought up about the comparison of colors.

### 4.3. Color Effects

Clark and Cena [44] studied insulation and color effects for heat transfer through clothing under radiation and showed that heat flux through black material was four times higher than through white material. Davis et al. [45] pointed out the influence of the dye. In a similar way, a study from Sweden [46] showed that under solar radiation (around 1 kW and a strong UV component) the darkness of the dye made the difference and not the color itself. The measurements within the Thermprotect project [21,22,23] showed that fiber reflectivity had an impact: more reflective orange Nomex^®^ absorbed less than matt natural white (slightly beige) cotton. Moreover, some studies showed that a camouflage black with reflective granules in the coating (also against IR cameras; similar to the Swiss-made ColdBlack^®^ textile) had less absorbance of heat than the green shades in the pattern [46]. The present study did not observe significant differences between colors at 1.5 and 2 kW/m^2^, while at 2.5 kW/m^2^, the exposure time in blue protective gear was significantly longer than in the red one. However, this should not lead to any long-going conclusions, as the number of test persons in both groups was small and individual characteristics and clothing fit seemed to dominate over possible, but small, color effects (Table 1 and Table 6).

### 4.4. Relation to Studies on Firefighter Protective Clothing

Several studies have been performed on firefighters’ protective clothing and the effect of air gap thickness on thermal protection. Air gap studies with firefighters’ clothing are a bit different relative to operators’ protective clothing. Firefighters’ protective clothing usually consists of a multilayer fabric with three protective layers, while operators’ clothing consists of one layer of protective clothing. Su et al. [13] investigated the effect of air gap thickness on the thermal protection of firefighters’ protective clothing against thermal radiation and hot steam with a testing apparatus for the thermal protective performance of clothing. They did not find an optimum air gap. They suggested that the air gap increase could reduce the heat flux but did not find a significant correlation between air gap thickness and the final heat flux. Ghazy [15] also reported the effect of air gaps entrapped in firefighters’ clothing. He stated that most of the models ignore the air gaps between multiple clothing layers, which play a crucial role in heat protection. Not modeling the air gaps in the clothing could underestimate the protective performance of the clothing. This was also studied with a bench-scale test apparatus in the study of Fu et al. [12]. They also concluded that the time before burn injuries increases with the increase in the air gap and provided insight into the contribution of the location of the air layer.

Current firefighting clothing probably offers sufficient protection against higher heat radiation levels than the currently stated criterion of 4.6 kW/m^2^ [25]. On the basis of the analysis of reports from the past, in which test persons in firefighting clothes were exposed to 7 kW/m^2^ for a maximum of 2 min, it was found in the year 2000 that almost all types of firefighting clothes complied with this [18]. Since then, the insulating value of firefighting clothing has only increased [1]. This means that the exposure duration limit at the critical radiation of 6.3 kW/m^2^ is probably equal to or longer than 3 min instead of the maximum of 2 min that is set by today’s recommendations [25]. However, this suggestion needs confirmation by a dedicated study.

After reaching a skin temperature of 35 °C, it often takes a few minutes before the skin temperature has reached the critical limit of 43 °C. Below the critical temperature of 43 °C, no skin burns can occur without a limiting time. Above this critical skin temperature, an inverse relationship between time and temperature exists [39]. This indicates that the process operators’ clothing can be suitable at a radiation intensity of 2.5 kW/m^2^ for very short exposure times only.

## 5. Conclusions and Recommendations

### 5.1. Conclusions

The following conclusions can be drawn on the basis of the present study and the analysis of previously published research into the heat protection of operators’ and firefighters’ protective clothing:

- EN ISO 11612 [29] certified process operator clothing offers sufficient protection to prevent skin burns during short-term activities up to 5 min at radiation intensities of 1.5 kW/m^2^—provided that the clothing fit is not tight and the correct size is used.
- For up to 3 min, the clothing offers sufficient protection against a radiation intensity of 2.0 kW/m^2^, provided that the clothing is not tight and the correct size is used.
- Supplementary (long) underwear and sufficiently loose clothing contribute to the protection of operators during an increased time at the earlier mentioned radiation levels or against higher heat radiation levels.
- Without supplementary underclothing at higher radiation intensities than 2.0 kW/m^2^, EN ISO 11612 [29] clothing is unsuitable for wearing as protective clothing.
- The maximum radiation levels for operators’ clothing set in the “Guidelines for maximum permissible levels of heat radiation for short deployment (maximum 5 min) of (company) firefighting personnel and operators at industrial companies” can be adjusted from 1 kW/m^2^ to 1.5 kW/m^2^ based on this validation study, provided fitting conditions are met.

Air gaps between the body and process operators’ protective clothing play an important role in the thermal protective performance of clothing. Smaller or no air gaps cause lower exposure durations and a higher rate of skin temperature increase at radiation levels between 1.5 and 2.0 kW/m^2^. This study indicates the relevance of stable air gaps in combination with body movements, which could be considered in the design of thermal protective clothing. Further research needs to be conducted on the effect of air gaps at higher radiation intensities and realistic body movements.

### 5.2. Recommendations

This validation study on the protective properties of process operators’ personal protective clothing provided important information to further specify the current guidelines for work at maximum acceptable heat radiation levels. Similar research on protective clothing for firefighters has not taken place since the year 2000. As the protective properties of firefighting clothing have significantly improved over the last two decades with regard to heat protection, it is recommended to conduct similar research with higher radiation levels for firefighting clothing.

Furthermore, it is recommended that during (planned) practice moments with heat flux control, skin temperature measurements should be taken from the candidates in order to test the current simulated experiments in the climate chamber.

It is recommended that the process operators avoid tight-fitting protective clothing. It is also recommended to ask operators in charge of possible incident responses to wear long underwear so that they are better protected.

## Figures and Tables

**Table 1:** Characteristics of test persons (TP).

TP	Gender	Age (Years)	Height (cm)	Weight (kg)	BMI	Clothing Color
1	Female	19	176.0	80.4	26.0	Blue
2	Male	58	186.0	99.3	28.7	Blue
3	Male	43	171.0	87.0	29.8	Blue
4	Male	58	176.0	85.8	27.7	Blue
5	Male	29	198.0	118.0	30.1	Blue
6	Male	38	178.5	103.8	32.6	Red
7	Male	50	179.6	83.0	25.7	Blue
8	Male	39	185.0	89.5	26.2	Red
9	Male	31	175.5	90.2	29.3	Red
10	Male	21	183.0	78.7	23.5	Red
11	Male	27	186.0	112.0	32.4	Red
12	Male	42	183.0	89.7	26.8	Blue
Average		38 ± 13	181.5 ± 7.1	93.1 ± 12.5	28.2 ± 2.7	

**Table 2:** Subjective responses scales.

Thermal Sensation		Comfort Sensation		Pain Sensation		Perceived Exertion	
−4	Very cold	0	Neutral	0	No pain	6	No exertion at all
−3	Cold	1	Slightly uncomfortable	1	Slightly painful	7	Extremely light
−2	Cool	2	Uncomfortable	2	Painful	8	
−1	Slightly cool	3	Very uncomfortable	3	Very painful	9	Very light
0	Neither warm nor cold	4	Very, very uncomfortable	4	Very, very painful	10	
1	Slightly warm					11	Light
2	Warm					12	
3	Hot					13	Somewhat hard
4	Very hot					14	
						15	Hard (heavy)
						16	
						17	Very hard
						18	
						19	Extremely hard
						20	Maximal exertion

**Table 3:** The percentiles of the tested population whose skin temperature stayed under 43 °C for 1, 3, and 5 min under the tested radiation intensities. In brackets is given the actual number of test persons who managed the given times.

Time Limit (min)	1.5 kW/m^2^	2 kW/m^2^	2.5 kW/m^2^
1	100 (12)	100 (12)	92 (11)
3	92 (11)	50 (6)	33 (4)
5	83 (10)	17 (2)	8 (1)

**Table 4:** The mean ∆Tskin/t in °C/s, with standard deviation, was measured at three radiation levels at 5 body locations. Calculated over time (t = 0 until the end of exposure to the radiation panel).

Skin Location	1.5 kW/m^2^	2 kW/m^2^	2.5 kW/m^2^
Chest	0.0121 ± 0.0028	0.0158 ± 0.0073	0.0202 ± 0.0132
Upper arm	0.0236 ± 0.0101	0.0400 ± 0.0274	0.0463 ± 0.0360
Abdomen	0.0328 ± 0.0172	0.0531 ± 0.0338	0.0777 ± 0.0433
Thigh	0.0379 ± 0.0196	0.0655 ± 0.0366	0.0978 ± 0.0616
Knee	0.0313 ± 0.0133	0.0520 ± 0.0318	0.0808 ± 0.0513

**Table 5:** Subjective responses at the start and at the end of each exposure to various radiation intensities (mean ± SD).

	1.5 kW/m^2^		2 kW/m^2^		2.5 kW/m^2^	
Responses	Start	End	Start	End	Start	End
Thermal sensation	0.0 ± 1.0	1.8 ± 0.7	−0.1 ± 0.5	2.0 ± 0.7	0.0 ± 0.7	2.6 ± 0.8
Comfort	0.1 ± 0.3	1.9 ± 0.9	0.0 ± 0.0	2.2 ± 0.7	0.2 ± 0.4	2.6 ± 1.1
Pain	0.0 ± 0.0	0.5 ± 0.7	0.0 ± 0.0	0.6 ± 0.8	0.0 ± 0.0	1.1 ± 1.1
RPE	6.1 ± 0.3	10.0 ± 2.2	6.2 ± 0.4	9.8 ± 2.1	6.3 ± 0.7	10.7 ± 2.6

**Table 6:** Air gap between body and clothing estimated at four different body locations in mm. The overall air gap is the mean of the air gaps of all body locations including the standard deviation. The air gap of the jacket is the mean air gap of the chest, upper arm, and abdomen including the standard deviation.

TP	Chest	Upper Arm	Abdomen	Thigh	Overall	Jacket
1	15.9	23.9	20.7	19.1	19.9 ± 3.3	20.2 ± 4.0
2	27.1	23.9	35.0	15.9	25.5 ± 7.9	28.6 ± 5.7
3	18.3	17.5	14.3	25.5	18.9 ± 4.7	16.7 ± 2.1
4	9.5	26.3	37.4	28.6	25.5 ± 11.6	24.4 ± 14.0
5	31.0	9.5	0.0	3.2	10.9 ± 14.0	13.5 ± 15.9
6	28.6	20.7	31.0	27.1	26.9 ± 4.4	26.8 ± 5.4
7	31.8	24.7	31.0	38.2	31.4 ± 5.5	29.2 ± 3.9
8	35.0	19.9	27.1	25.5	26.9 ± 6.2	27.3 ± 7.6
9	35.0	25.5	40.6	33.4	33.6 ± 6.2	33.7 ± 7.6
10	63.7	27.1	60.5	36.6	47.0 ± 17.9	50.4 ± 20.3
11	36.6	17.5	14.3	27.1	23.9 ± 10.1	22.8 ± 12.1
12	19.1	20.7	23.9	24.7	22.1 ± 2.6	21.2 ± 2.4
Average	29.3 ± 13.9	21.4 ± 5.0	28.0 ± 15.4	25.4 ± 9.5	26.0 ± 11.7	26.2 ± 12.4

**Table 7:** Differences between tight, regular, and loose clothing fit group means in exposure time.

Groups Based on	1.5 kW/m^2^	2 kW/m^2^	2.5 kW/m^2^
A Chest	F(2,9) = 0.544, *p* = 0.599 **	F(2,9) = 0.089, *p* = 0.916	F(2,9) = 1.742, *p* = 0.229
B Upper arm	F(2,9) = 2.749, *p* = 0.117 **	F(2,9) = 0.929, *p* = 0.430	F(2,9) = 1.156, *p* = 0.357
C Abdomen	F(2,9) = 2.749, *p* = 0.117 **	F(2,9) = 6.445, *p* = 0.018 * Posthoc: significant difference between tight fit and loose fit (*p* = 0.018)	F(2,9) = 0.260, *p* = 0.777
D Thigh	F(2,9) = 0.544, *p* = 0.599 **	F(2,9) = 0.513, *p* = 0.615	F(2,9) = 1.153, *p* = 0.358
E Jacket	F(2,9) = 0.544, *p* = 0.599 **	F(2,9) = 1.671, *p* = 0.241	F(2,9) = 0.539, *p* = 0.601
F Whole suit	F(2,9) = 0.544, *p* = 0.599 **	F(2,9) = 1.671, *p* = 0.241	F(2,9) = 0.312, *p* = 0.739

* Significant *p* < 0.05, ** Note: violated homogeneity of variances. Some groups do not show variance, because 10 of 12 participants reached the maximum exposure time of 300 s at the radiation level of 1.5 kW/m^2^.

**Table 8:** Simple linear regression outcomes, *p*-values. Different air gaps as the independent variable and exposure time or body location specific ∆Tskin/t as the dependent variable. Regressions were made for all three different radiation intensities.

	Radiation Intensity (kW/m^2^)								
Air Gap at	1.5	2.0	2.5	1.5	2.0	2.5	1.5	2.0	2.5
	1.5	2.0	2.5	1.5	2.0	2.5	1.5	2.0	2.5
	**Exposure Time**			**∆Tskin/t: Mean Whole Body**			**∆Tskin/t: Mean Upper Body**		
Chest	0.692	0.601	0.365	0.928	0.948	0.612	0.664	0.682	0.321
Upper arm	0.002 *	0.022 *	0.181	0.001 *	0.003 *	0.043 *	0.001 *	0.005 *	0.128
Abdomen	0.019 *	0.010 *	0.452	0.011 *	0.011 *	0.106	0.033 *	0.042 *	0.315
Thigh/trousers	0.034 *	0.255	0.073	0.024 *	0.126	0.148	-	-	-
Mean jacket	0.156	0.050 *	0.847	0.091	0.094	0.363	0.189	0.214	0.756
Mean whole suit	0.087	0.058	0.549	0.048 *	0.076	0.266	0.144	0.206	0.650
	**∆Tskin/t: mean lower body**			**∆Tskin/t: chest**			**∆Tskin/t: upper arm**		
Chest	-	-	-	0.533	0.120	0.482	-	-	-
Upper arm	-	-	-	-	-	-	0.335	0.215	0.317
Abdomen	-	-	-	-	-	-	-	-	-
Thigh/trousers	0.013 *	0.066	0.072	-	-	-	-	-	-
Mean jacket	-	-	-	0.085	0.010 *	0.057	0.806	0.456	0.722
Mean whole suit	0.040 *	0.043 *	0.138	0.059	0.012 *	0.083	0.933	0.555	0.783
	**∆Tskin/t: abdomen**			**∆Tskin/t: thigh**			**∆Tskin/t: knee**		
Chest	-	-	-	-	-	-	-	-	-
Upper arm	-	-	-	-	-	-	-	-	-
Abdomen	0.011 *	0.017 *	0.162	-	-	-	-	-	-
Thigh/trousers	-	-	-	0.022 *	0.117	0.136	0.009 *	0.041 *	0.053
Mean jacket	0.072	0.088	0.475	-	-	-	-	-	-
Mean whole suit	0.030 *	0.059	0.331	0.036 *	0.056	0.156	0.059	0.046 *	0.172

* Significant *p* < 0.05.

## Data Availability

Not applicable.
